# The remarkable tenacity of sulfadoxine-pyrimethamine

**DOI:** 10.1016/S1473-3099(18)30796-5

**Published:** 2019-03-25

**Authors:** Titus H Divala, Lauren M Cohee, Miriam K Laufer

**Affiliations:** University of Malawi College of Medicine, Blantyre, Malawi (THD); Center for Vaccine Development and Global Health, University of Maryland School of Medicine, Baltimore, MD 21201, USA (LMC, MKL)

*Plasmodium falciparum* infection during pregnancy causes low birthweight in the newborn, which is a risk factor for infant mortality.^1^ Intermittent preventive treatment in pregnancy (IPTp) with sulfadoxine-pyrimethamine was introduced with the primary aim of ameliorating the effect of malaria on birthweight. At the time of its introduction 15–20 years ago, many countries were still using sulfadoxine-pyrimethamine as the first-line treatment for malaria. Concerns immediately arose about the spread of sulfadoxine-pyrimethamine-resistant malaria and the effect of resistance on the effectiveness of the drug combination for both the treatment and prevention of malaria. Over the past decade, all countries in sub-Saharan Africa have changed their first-line treatment of malaria to an artemisinin-based combination therapy, raising the possibility that sulfadoxine-pyrimethamine resistance might begin to decrease in prevalence. However, in contrast to the re-emergence of chloroquine-susceptible malaria after the removal of drug pressure,^2^ sulfadoxine-pyrimethamine resistance seems to be fixed in the parasite population in Africa, despite its withdrawal from use as the first-line treatment for uncomplicated disease.^3^ This fixation might be due to the use of sulfadoxine-pyrimethamine for IPTp, or the frequent use of another antifolate drug combination, co-trimoxazole (trimethoprimsulfamethoxazole), for the treatment of bacterial infection and for prophylaxis among people living with HIV. Regardless of the mechanism of its persistence, sulfadoxine-pyrimethamine resistance seems to be here to stay.

In *The Lancet Infectious Diseases*, Anna Maria van Eijk and colleagues^4^ report results of a meta-analysis of aggregated-data from 57 studies (involving 59 457 births) and of individual-patient data from 13 surveys (42 394 births), assessing the effect of sulfadoxine-pyrimethamine resistance on sulfadoxinepyrimethamine IPTp effectiveness. van Eijk and colleagues collected data on molecular markers of sulfadoxine-pyrimethamine resistance from published reports, individual authors, and population prevalence maps compiled by the Worldwide Antimalarial Resistance Network (WWARN). Data on transmission intensity were collected from the Malaria Atlas Project. The investigators categorised study areas as having low, moderate, or high resistance by exploring various thresholds of prevalence of three mutations in the *P falciparum dhps* gene: Lys540Glu, Ala437Gly, and Ala581Gly. The distribution of sulfadoxine-pyrimethamine-resistant genotypes differed: in east and southern Africa, the high prevalence of *dhps* Lys540Glu defined high-level resistance to sulfadoxine-pyrimethamine, but was rarely identified in central and west Africa, where *dhps* Ala437Gly prevalence distinguished low from moderate levels of resistance. Pooled estimates from the aggregated-data meta-analysis suggested that protection from low birthweight decreases with increasing prevalence of *dhps* Lys540Glu (P_trend_=0·0060), but is unaffected by the prevalence of *dhps* Ala437Gly mutations (P_trend_=0·35). The individual-participant analysis of survey data, however, shows the resilience of sulfadoxine-pyrimethamine IPTp, with a protective effect even in areas with a *dhps* Lys540Glu prevalence of more than 90% and Ala581Gly prevalence of less than 10% (relative risk reduction [RRR] 10% [95% CI 7–12%]). This protection waned in areas with both a high prevalence of *dhps* Lys540Glu and a *dhps* Ala581Gly prevalence of 10% or higher (RRR 0·5% [−16 to 14]), but this most extreme resistant genotype was only found in a small number of locations in east Africa.

The need for such a large meta-analysis to link sulfadoxine-pyrimethamine resistance to its protective efficacy against low birthweight has been clear for many years. Although the failure of sulfadoxine-pyrimethamine to treat and prevent malaria infection during pregnancy has been documented by numerous studies,^5^ any effect of the decreased antimalarial efficacy of sulfadoxine-pyrimethamine with respect to birthweight has been difficult to detect. Given the challenges of conducting clinical trials in pregnant women^6^ and the variable attributable fraction of malaria to low birthweight,^7^ this report shows the catalytic impact that data-sharing platforms, such as those established by WWARN and the Malaria Atlas Project, can have to translate research data into evidence to inform public health.

The results of this study have important implications for the types of data that policy makers require to predict the efficacy of sulfadoxine-pyrimethamine IPTp. In low-resource settings, it might be most beneficial to limit molecular testing to *dhps* Ala581Gly in east and southern Africa and to include *dhps* Ala437Gly in west and central Africa. However, to accurately model the future risk of widespread sulfadoxine-pyrimethamine IPTp failure, we must better understand how resistance emerges and the factors that drive parasite gene flow across geo graphic regions. Why are quintuple-mutant sulfadoxine-pyrimethamine-resistant parasites so rare in west and central Africa? Why is the highly resistant *dhps* Ala581Gly present in only a few settings and only in the context of the quintuple mutant? What conditions favour or discourage the emergence and spread of sulfadoxine-pyrimethamine-resistant parasites? Previous experience does not provide clear answers to these questions. Although chloroquine-resistant malaria emerged a few times in distinct geographic locations, the genomic analysis of the spread of artemisinin-resistant malaria indicates both independent emergence and also geographic spread.^8^ The combination of the types of integrated international database collaborations described by van Eijk and colleagues, along with new methods in genomics and modelling, will allow us to not only track the failure of drugs, but also to be proactive in developing interventions to limit the spread and impact of antimalarial drug resistance.

## References

[1] Eisele TP, Larsen DA, Anglewicz PA, Malaria prevention in pregnancy, birthweight, and neonatal mortality: a meta-analysis of 32 national cross-sectional datasets in Africa. Lancet Infect Dis 2012; 12: 942–49. 22995852 10.1016/S1473-3099(12)70222-0

[2] Frosch AEP, Laufer MK, Mathanga DP, Return of widespread chloroquine-sensitive *Plasmodium falciparum* to Malawi. J Infect Dis 2014; 210: 1110–14. 24723474 10.1093/infdis/jiu216 PMC6281358

[3] Artimovich E, Schneider K, Taylor TE, Persistence of sulfadoxine-pyrimethamine resistance despite reduction of drug pressure in Malawi. J Infect Dis 2015; 212: 694–701. 25672905 10.1093/infdis/jiv078 PMC4539899

[4] van Eijk AM, Larsen DA, Kayentao K. Effect of *Plasmodium falciparum* sulfadoxine-pyrimethamine resistance on the effectiveness of intermittent preventive therapy for malaria in pregnancy in Africa: a systematic review and meta-analysis. Lancet Infect Dis 2019; published online March 25. 10.1016/S1473-3099(18)30732-1. 30922818

[5] Desai M, Gutman J, Taylor SM, Impact of sulfadoxine-pyrimethamine resistance on effectiveness of intermittent preventive therapy for malaria in pregnancy at clearing infections and preventing low birth weight. Clin Infect Dis 2016; 62: 323–33. 26486699 10.1093/cid/civ881 PMC4762476

[6] Divala TH, Mungwira RG, Laufer MK. Moving targets: the challenges of studying infectious diseases among pregnant women in resource limited settings. Vaccine 2015; 33: 6401–05. 26319059 10.1016/j.vaccine.2015.08.042 PMC4920047

[7] Walker PG, ter Kuile FO, Garske T, Menendez C, Ghani AC. Estimated risk of placental infection and low birthweight attributable to Plasmodium falciparum malaria in Africa in 2010: a modelling study. Lancet Glob Health 2014; 2: e460–67. 25103519 10.1016/S2214-109X(14)70256-6

[8] Takala-Harrison S, Laufer MK. Antimalarial drug resistance in Africa: key lessons for the future. Ann NY Acad Sci 2015; 1342: 62–67. 25891142 10.1111/nyas.12766 PMC4527866

